# Study on Extraction Process of Root of Henry Wood Betony Polysaccharides and Their Antitumor Activity against S180

**DOI:** 10.3390/molecules26082359

**Published:** 2021-04-19

**Authors:** Haibo Feng, Lan Tian

**Affiliations:** 1 Animal Husbandry and Veterinary Medicine, Southwest Minzu University, Chengdu 610041, China; 2 Key Laboratory of Ministry of Education and Sichuan Province for Qinghai-Tibetan Plateau Animal Genetic Resource Reservation and Utilization, Chengdu 610041, China; 3 Department of Veterinary Medicine, Southwest University, Chongqing 402460, China; slimrecovery@163.com

**Keywords:** polysaccharides of root of Henry wood betony, anti-tumor activity, cell apoptosis, immunomodulator, S180

## Abstract

We optimized the hot water extraction of polysaccharides from the root of Henry wood betony (RHWPs) using a uniform test and explored their anti-tumor activities in vitro and in vivo. The optimal extraction conditions were as follows: 40 min extraction time, liquid/solid ratio 30 mL/g, 100 min soaking time, two extraction cycles, 100% ethanol concentration, and extraction temperature of 80 °C. The molecular weight distribution of RHWPs with MWs was 228,600 g/mol and 5001 g/mol. The IR spectrum further indicated that RHWPs are acidic polysaccharides containing pyranose and furan rings. The main monosaccharides found in RHWPs were mannose, ribose, l-rhamnose monohydrate, glucuronic acid, galacturonic acid, glucose, galactose, xylose, arabinose, and fucose. RHWPs inhibited the proliferation of S180 tumor cells and induced apoptosis in vitro. Oral administration of RHWPs to tumor-bearing mice significantly inhibited the growth of the S180 xenografts, accelerated apoptosis in tumor cells, and expanded the necrotic regions. Furthermore, RHWPs also markedly increased the levels of TNF-α, IFN-γ, and IL-2 in the sera of tumor-bearing mice, and activated immune cells such as lymphocytes, NK cells, and macrophages, thereby inducing tumor cell apoptosis. Taken together, RHWPs are a promising anti-tumor agent that ought to be explored further.

## 1. Introduction

According to the World Health Organization (WHO), cancer is the second leading cause of death worldwide and was responsible for 9.6 million deaths in 2018, corresponding to one in six individuals globally [1]. The combination of chemotherapy with surgery and radiotherapy has successfully improved the clinical outcomes of several cancers. However, potent adverse effects and drug resistance have blunted the efficacy of the current oncotherapies, which calls for the development of novel drugs [2]. Natural bioactive compounds with anti-tumor action have garnered considerable attention in recent years on account of their higher efficacy, lower toxicity, and immunomodulatory effects. In addition, compared to chemotherapeutic drugs, natural products target multiple pathways and effectors [3], which can significantly broaden their clinical applications.

Polysaccharides, one of the four basic biomolecules that constitute life, have anti-tumor, pro-immunity, hypoglycemic, antioxidant, anti-virus, and other pharmacological effects [4]. Studies show that the anti-tumor effects of plant-derived polysaccharides can be attributed to three main mechanisms: prevention of malignant transformation, augmentation of the anti-tumor immune response, and direct killing tumor cells through apoptosis induction [5]. Ikekawa et al. found that dietary supplementation with polysaccharides from Flammulina velutipes and Agaricus blazei significantly decreased the number of tumor-bearing mice, indicating a preventive effect of these polysaccharides [6].

The pro-immune effects of polysaccharides include activation of effector cells such as monocytes, macrophages, T and B lymphocytes, and natural killer (NK) cells; increased phagocytosis of the reticuloendothelial system; and induction of cytokines such as tumor necrosis factor (TNF), interleukin-1 (IL-1), interferon (IFN), and IL-2. Administration of Achyranthis bidentata polysaccharides increased the weight of spleen and thymus and delayed their chemo/radiotherapy-induced functional decline in immunosuppressed mice. In addition, the polysaccharides also improved the carbon particle and foreign body clearance index by enhancing phagocytic function and promoted lymphocyte proliferation [7]. Likewise, the saponins and polysaccharide of Panax japonicus increased the spleen index of immunosuppressed mice, enhanced the proportion of B lymphocytes and NK cells, and promoted IFN-γ and IL-2 secretion [8]. The polysaccharides from pomegranate rind also increased thymus and spleen weight in normal mice and enhanced the phagocytic ability of macrophages [9], whereas Radix glehniae polysaccharides significantly enhanced splenic NK cell activity, T lymphocyte expansion, and serum immunoglobulin levels in hyperthyroid mice [10]. Furthermore, ginseng polysaccharides can inhibit the proliferation of murine sarcoma S180 cells in vivo by increasing host immunity, inhibiting tumor cell proliferation, and inducing tumor cell apoptosis [11]. Similar anti-tumor effects have also been reported for Codonopsis pilosula polysaccharides against the colon cancer cell line HCT116 [12]. Ganoderma atrum polysaccharides augmented the host immune response in tumor-bearing mice by activating peritoneal macrophages through TLR4 receptor signaling and promoted secretion of TNF-α via the NF-κB and the p38 MAPK pathways [3].

Ganoderma lucidum polysaccharides can directly inhibit tumor cell growth by activating the cAMP/PKA signaling pathway via DG-PKC downregulation [13] and triggering the mitochondrial apoptosis cascade [3]. Tumor cells incubated with these polysaccharides showed cell cycle arrest and higher apoptosis rates [14,15,16]. Furthermore, protein-bound polysaccharides isolated from mulberry inhibited proliferation of SW480 human colon cancer cells [15], Lycium barbarum polysaccharides induced S phase cell cycle arrest and apoptosis in human hepatoma QGY7703 cells [17], and the carboxymethylated polysaccharide of Pleurotus tuber-regium also blocked cell cycle progression and triggered apoptosis in MCF-7 cells [5].

Henry wood betony, also known as chasing wind arrow and cock flower root, is a perennial herb belonging to the Artemisia Scrophulariaceae family. It is widespread across Yunnan Province in China, and grows wild in the hillsides, grasslands, and forests of Jiangsu, Jiangxi, Hunan, Yunnan, Guizhou, Guangxi, Guangdong, etc. The roots of this herb (RHW) have been described in the “National Chinese Herbal Medicine Compilation” as warm, sweet, and slightly bitter. It has the effects of tonifying qi and blood, passing through meridians, and relieving cough and asthma, and it is used to treat dizziness, tinnitus, shortness of breath, shortness of muscles, and bronchitis. The “Kunming Folk Common Herbs” has recorded that RHW can also be used to treat smoldering.

In the present study, we extracted RHWPs using hot water and analyzed their anti-tumor effects in vitro and in vivo. The RHWP extract inhibited the proliferation of murine sarcoma 180 (S180) cells and induced apoptosis, and moreover significantly retarded S180 xenograft growth in a mouse model. In addition, RHWP showed an immunomodulatory effect in the tumor-bearing mice by activating immune effector cells and increasing cytokine secretion.

## 2. Results and Discussion

### 2.1. Single-Factor Experiment Analysis

**Figure 1:**
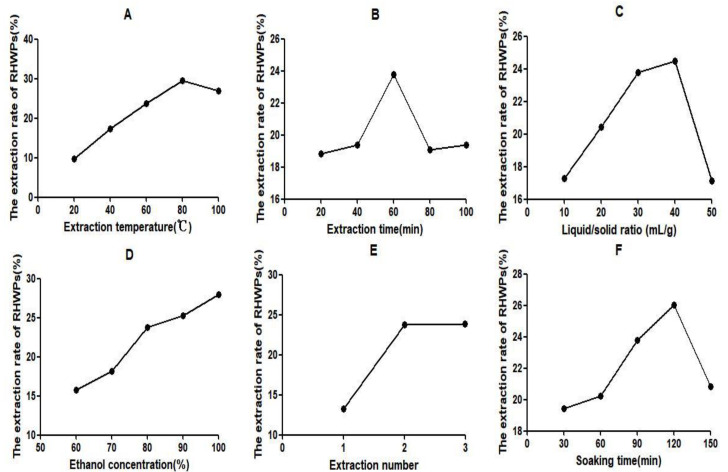
Effects of different extraction temperatures (**A**), extraction time (**B**), liquid/solid ratio (**C**), ethanol concentration (**D**), extraction number (**E**), and soaking time (**F**) on the extraction rate of RHWPs.

The single-factor test results of RHWPs are shown in Figure 1. The extraction temperature of 80 °C, 60 min extraction time, 40 mL/g liquid/solid ratio, 100% ethanol concentration, two extraction cycles, and soaking time of 120 min achieved optimal extraction rate and minimal energy consumption.

### 2.2. Optimization of Procedure by Uniform Design

The uniform experimental results are shown in Table 1, and the analysis results in Table 2. The data were subjected to multiple linear regression analysis using SPSS 22.0 to obtain a linear regression equation Y = 57.638 − 0.053B − 0.081C − 0.118D, with S = 0.379, R = 0.995, and F = 0.014. The regression equation was significant at a = 0.005, and the theoretical prediction of Y was 36.069 ± 2.482%, and its range was 33.031–39.167%. The regression equation and analysis of variance showed that the order of factors affecting the extraction effect was B > C > D. According to the results from uniform design optimization, the optimized RHWPs extraction parameters were as follows: extraction temperature of 80 °C, 40 min extraction time, liquid/solid ratio 30 mL/g, and soaking time of 100 min. To verify the results of uniform design, we extracted RHWPs under these conditions, and obtained a yield of 36.74 ± 3.18%. It is consistent with the predicted extraction rate, thereby validating the stability and feasibility of the extraction conditions.

### 2.3. Characterization of the RHWPs

#### 2.3.1. Analysis of Physicochemical Properties

The total sugar content of RHWPs was 92.19% by using the Anthrone–sulfuric acid method. The RHWPs powder color was milky, and it was water-soluble, but no proteins, starch, polyphenols, or reducing sugar were present. Uronic acid content was determined to be 2.9%.

#### 2.3.2. FTIR Spectroscopy

**Figure 2:**
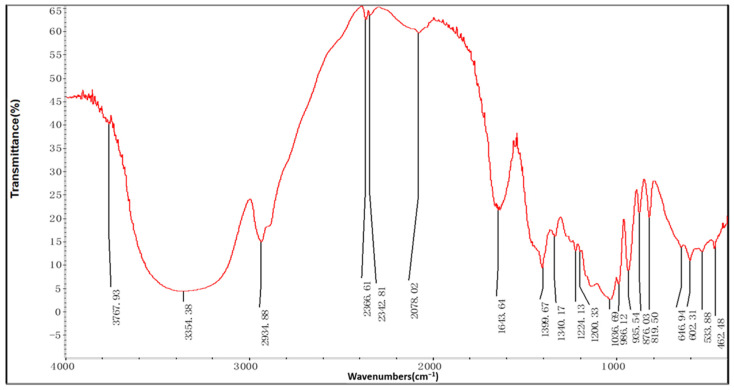
IR spectrum of RHWPs.

RHWPs were characterized by FTIR. As shown in Figure 2, the FTIR spectrum of RHWP fraction showed a broadly stretched intense peak at around 3360 cm^−1^ that corresponds to OH [18], weak absorption at 2937 cm^−1^ due to the C–H stretching vibration [19], and a relatively strong absorption peak at 1634 cm^−1^ indicating C=O [20]. In addition, the peaks from 950 cm^−1^ to 1200 cm^−1^ suggested the presence of C–O–C and C–OH bonds and hydroxyl of pyranose ring, all of which are indicative of pyranose [21,22]. Finally, a peak at 876 cm^−1^ corresponded to β-glycosidic bond, and that at 650 cm^−1^ is likely characteristic of rhamnose [23].

#### 2.3.3. GPC Analysis

**Figure 3:**
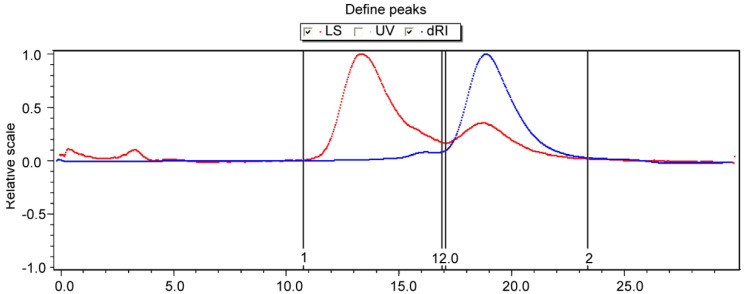
The GPC spectrum of RHWPs.

The GPC analysis of RHWPs is shown in Figure 3 and Table 3. Peak 1 is the red spectrum; the signal value was detected by the laser detector. Peak 2 is the blue spectrum; the signal value was detected by the refractive index detector. Two peaks at molecular weights of 228,600 g/mol and 5001 g/mol can be observed. The molecular weight distribution was wide with large PD value. PD indicates the shape of molecular weight distribution. When the PD value is closer to 1, the molecular weight distribution is narrower [24]. According to Table 3, the distribution of molar mass in peak 2 was more homogeneous than that in peak 1.

#### 2.3.4. Analysis of Monosaccharide Compositions

**Figure 4:**
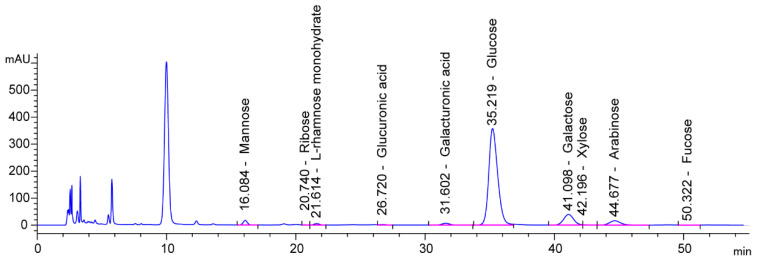
The monosaccharide compositions of RHWPs.

The compositions of monosaccharides in RHWPs are shown in Figure 4 and Table 4. The monosaccharides in RHWPs were found to be mannose, ribose, L-rhamnose monohydrate, glucuronic acid, galacturonic acid, glucose, galactose, xylose, arabinose, and fucose. Among them, glucose encompassed the largest proportion, followed by galactose and arabinose, and the other monosaccharides were found to be present only in relatively small quantities.

### 2.4. RHWPs Inhibited the Proliferation of S180 Cells and Induced Apoptosis In Vitro

As shown in Table 5, the RHWPs inhibited the growth of S180 cells in vitro in a dose-dependent manner. High concentration of 8 mg/mL increased the inhibition rate to 63.85%.

**Figure 5:**
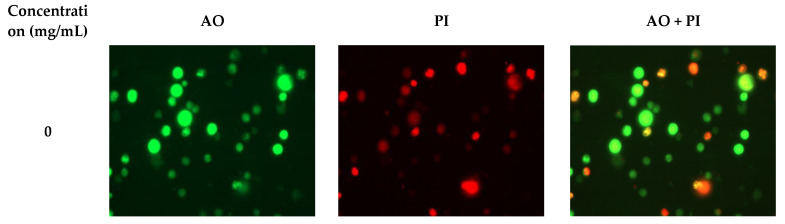

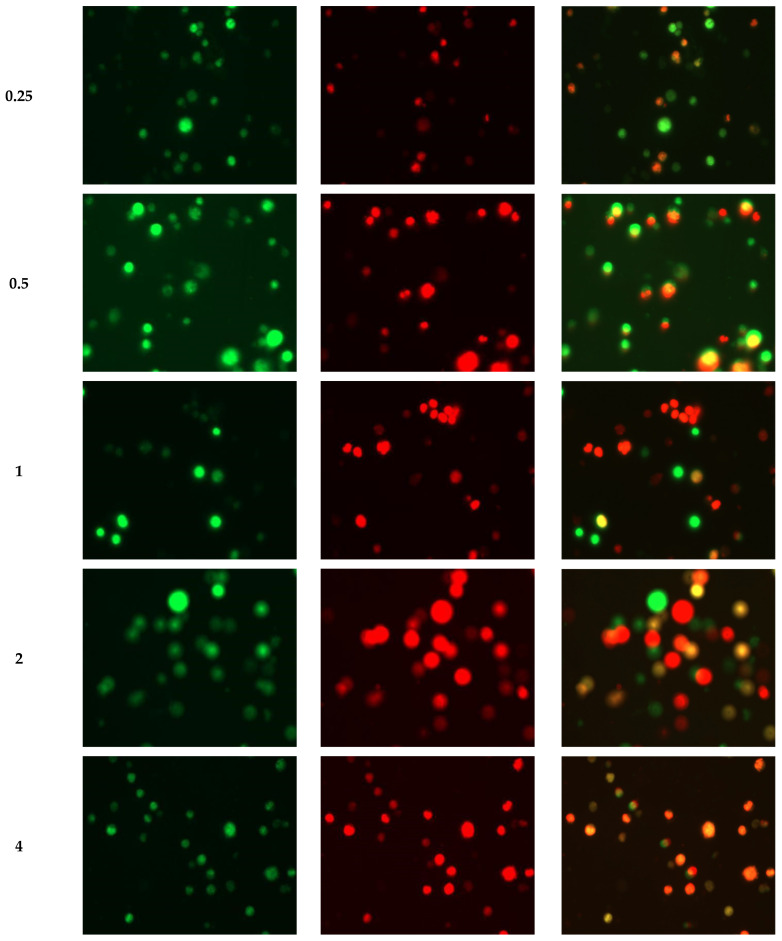
Effects of RHWPs on apoptotic morphological of S180 cells. S180 cells treated with the different concentration of RHWPs for 72 h. The cell suspension was stained with acridine orange (AO; 100 μg/mL) and propidium iodide (PI; 100 μg/mL). The stained cells were immediately observed under a fluorescence microscope. The live cells are green, apoptotic (orange), and necrotic (red) cells. Green fluorescence is the cells labeled by AO, while red fluorescence indicates the necrotic cells stained with PI. Orange fluorescence indicates apoptotic cells.

The apoptotic cells were detected through acridine orange (AO) and propidium iodide (PI) double staining to distinguish between live, apoptotic, and necrotic cells. AO passes through the cell membrane of viable cells and stains the nucleus green or yellow-green, while PI can only penetrate the damaged cell membranes of late apoptotic cells and dead cells, imparting an intense orange/red color. As shown in Figure 5, the untreated control cells were viable and retained their original shape, whereas RHWPs significantly increased the number of apoptotic and necrotic cells in a dose-dependent manner. In addition, the RHWP-treated cells showed shrinkage, which is also characteristic of late apoptosis. Taken together, RHWPs inhibited the proliferation of S180 cells and induced apoptosis.

### 2.5. RHWPs Inhibited S180 Ascite Tumor Growth and Expanded the Necrotic Regions

As shown in Table 6, RHWPs-Low decreased the tumor weight by 27.77% compared to that in the untreated control groups (*p <* 0.05), and the inhibitory effects of cyclophosphamide (CTX), RHWPs-Mid, and RHWPs-High were even greater (*p <* 0.01) at 72.79%, 63.63%, and 46.98%, respectively.

**Figure 6:**
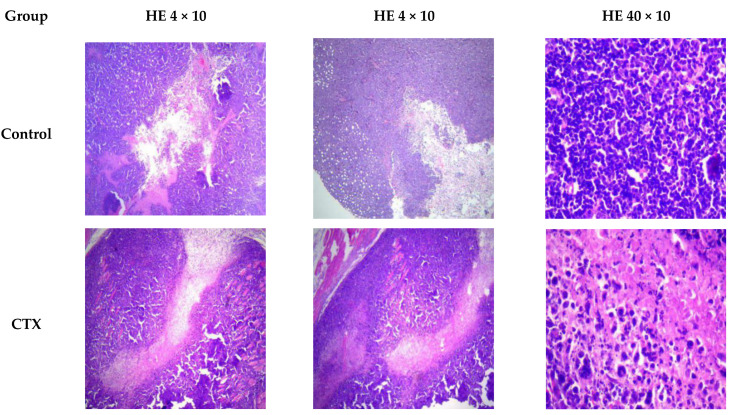

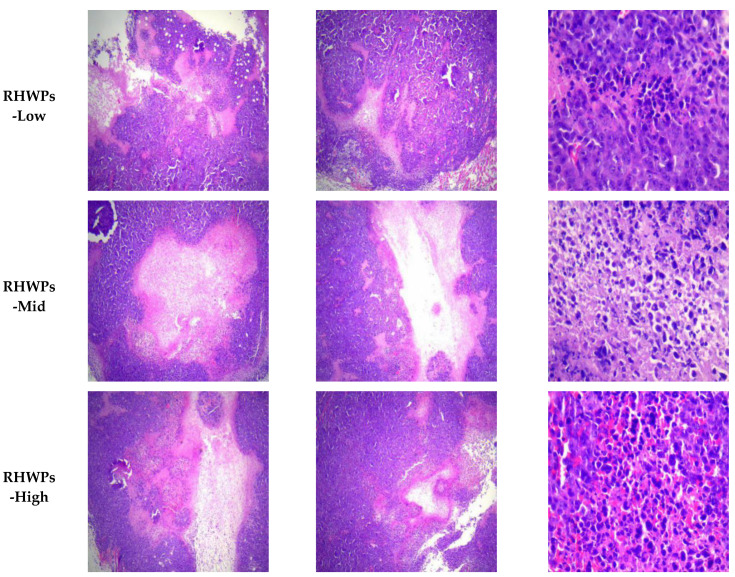
Representative images of H&E-stained tumor tissues of the indicated groups. Tumor tissue of different groups were collected 24 h after the last drug administration in order to investigate tumor tissue changes. The tissue sections were stained with hematoxylin and eosin and observed under a light microscope (UOP, USA) at 100× and 400× magnification to record the pathological changes. Different degrees of hyperplasia were found in different groups.

Furthermore, hematoxylin and eosin staining [25] showed extensive necrosis in the tumors of the CTX and the RHWP-treated mice (Figure 6). RHWPs-Mid (150 mg/kg) showed the optimal inhibitory effect, resulting in significantly reduced number of tumor cells with lighter staining, clear signs of lysis, and round shape. Thus, RHWPs exhibited significant anti-tumor activity in vivo.

### 2.6. RHWPs Increased Secretion of TNF-α, IFN-γ, and IL-2 in the Sera of Tumor-Bearing Mice

**Figure 7:**
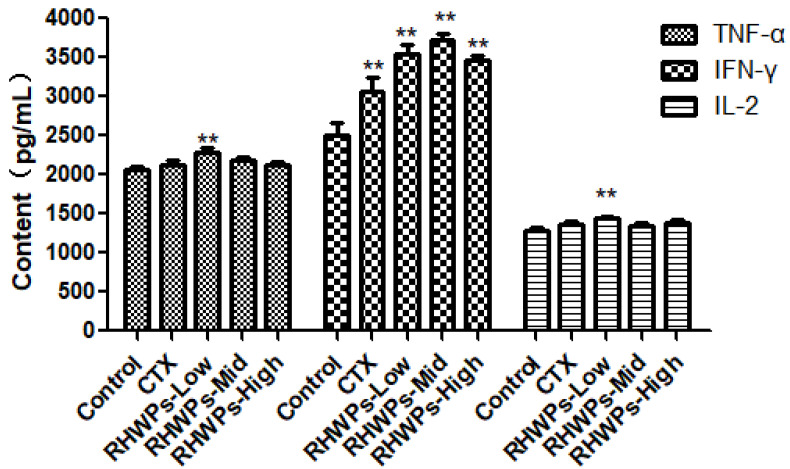
TNF-α, IFN-γ, and IL-2 levels in the sera of the mice. The concentration of TNF-α, IL-2, and IFN-γ in serum of ICR mice was determined by ELISA kit. Data are means ± SD (*n* = 10). Significant differences from the control groups are designated as ** *p* < 0.01.

Cytokines are key effectors of the immune response and play a major role in inhibiting tumor growth [26]. TNF-α is mainly secreted by monocytes, macrophages, and lymphocytes, and promotes the proliferation and differentiation of B and T lymphocytes, as well as tumor cell-killing effect of cytotoxic T lymphocytes and NK cells. It also has a direct inhibitory effect on tumor cells [27]. IFN-γ is primarily produced by T lymphocytes, NKT cells, NK cells, and other inflammatory cells. In addition, macrophages, dendritic cells (DC) and even tumor cells secrete IFN-γ. It plays a key role in promoting the anti-viral and anti-tumor immune responses [28], and also inhibits tumor angiogenesis, growth, and metastasis [29,30,31]. T cells are the major producers of IL-2, which promotes lymphocyte mitosis, enhances NK cell cytotoxicity, and increases antibody production [32]. As shown in Figure 7, serum TNF-α levels markedly increased in the RHWPs-Low group compared to the control (*p <* 0.01), whereas CTX and higher doses of RHWP did not have any significant effect (*p* > 0.05). In contrast, the circulating IFN-γ levels were significantly higher in the mice treated with CTX and all RHWP doses relative to the control group (*p <* 0.01). Finally, the levels of IL-2 were also considerably increased in the RHWPs-Low group compared to the control group (*p <* 0.01), whereas the other drugs did not significantly affect its serum content (*p* > 0.05). Taken together, RHWPs may inhibit tumor growth by promoting secretion of immunomodulatory cytokines.

### 2.7. RHWPs Increased Lymphocyte Proliferation in the Tumor-Bearing Mice

**Figure 8:**
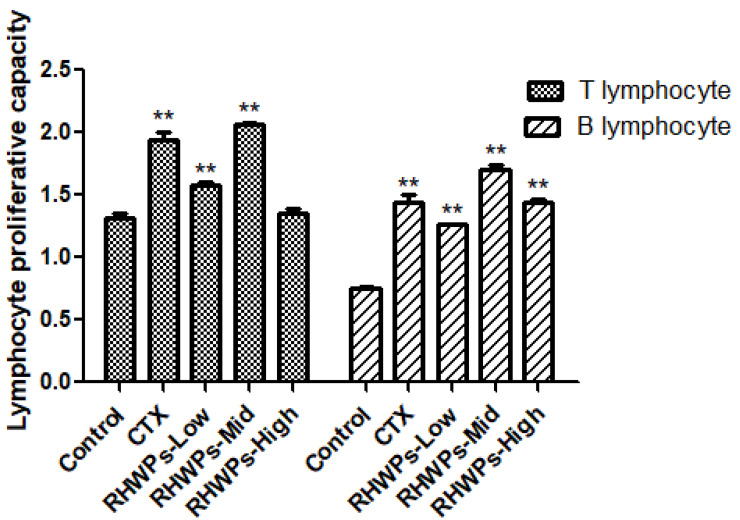
Effect of RHWPs on lymphocyte proliferation in S180 tumor-bearing mice. The splenocytes were isolated and co-cultured with ConA or LPS for 48 h, and the WST-8 approach was used to quantify proliferation. Data are means ± SD (*n* = 10), Significant differences from the control groups are designated as ** *p* < 0.01.

T cells are the major effectors of cellular immunity and mediate anti-tumor and anti-viral responses through direct cytotoxicity. In addition, they also promote humoral immunity by stimulating antibody production by B cells [26]. We analyzed the proliferative capacities of splenic B and T lymphocytes from the tumor-bearing mice by stimulating the cells with lipopolysaccharide (LPS) and concanavalin A (ConA), respectively [33]. As shown in Figure 8, CTX, RHWPs-Low, and RHWPs-Mid significantly increased the proliferative rate of T cells compared to the control (*p <* 0.01), whereas RHWPs-High had no significant effect (*p* > 0.05). B cell proliferation was significantly enhanced by all drugs, of which RHWPs-Mid had the optimal effect (*p* < 0.01). These findings further underscore the immunomodulatory role of RHWPs in the context of tumor inhibition.

### 2.8. RHWPs Enhanced the Effector Functions of NK Cells and Peritoneal Macrophages In Vivo

NK cells are important effectors of anti-tumor immune response [34]. As shown in Table 7, CTX, RHWPs-Low, RHWPs-Mid, and RHWPs-High significantly increased the tumor cell-killing activity of NK cells. The effect of CTX and RHWPs-High were the most potent, resulting in 39.71% and 29.78% killing activity, respectively.

**Figure 9:**
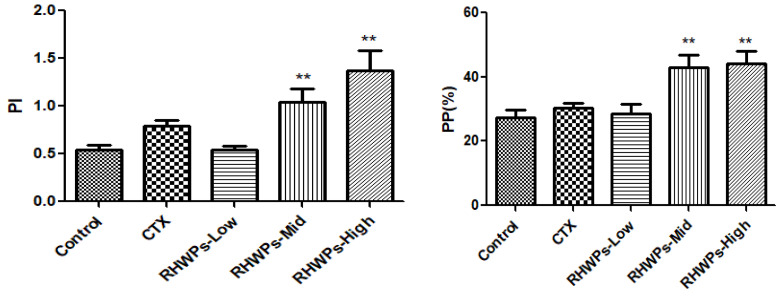
Effect of RHWPs on PP and PI in S180 tumor-bearing mice. The PP and PI are presented as mean ± SD (*n* = 10). Note: significant differences from the control groups are designated as ** *p* < 0.01.

Macrophages are the primary cellular component of the innate immune system, and are activated during infection, inflammation, and wound healing [35,36,37]. Macrophages phagocytose pathogens and other foreign bodies and secrete pro-inflammatory mediators such as nitric oxide (NO), prostaglandin E2 (PGE2), and cytokines (including TNF-α, IL-6, and IL-1β) [38]. As shown in Figure 9, RHWPs-Mid and RHWPs-High significantly increased the phagocytosis index and phagocytosis rate of peritoneal macrophages (*p <* 0.01 for both), while CTX and the RHWPs-Low failed to show any significant impact on either parameter (*p* > 0.05).

**Figure 10:**
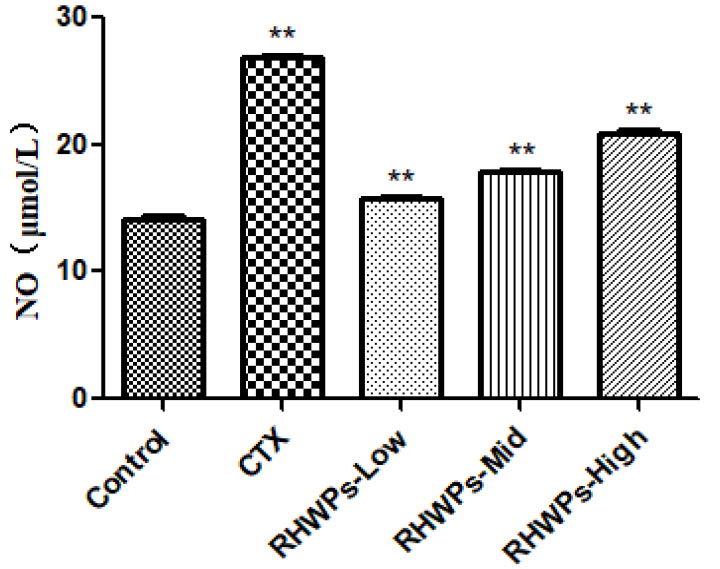
Effect of RHWPs on NO content in serum of S180 tumor-bearing mice. The concentration of NO in serum of S180 tumor-bearing mice was determined by ELISA kit. Data are means ± SD (*n* = 10). Significant differences from the control groups are designated as ** *p* < 0.01.

Consistent with the enhanced phagocytic function, the peritoneal macrophages isolated from the CTX-, RHWPs-Low-, RHWPs-Mid-, and RHWPs-High-treated mice produced a significantly higher amount of NO compared to the control (*p <* 0.01; Figure 10).

## 3. Experimental Section

### 3.1. Chemicals and Reagents

RHWs were purchased from the Sichuan Chengdu Medicinal Materials Market in China. S180 cells and YAC-1 cells were provided by Shanghai Zhong Qiao Xin Zhou Biotechnology Co. Ltd. (Shanghai, China). RPMI-1640 was purchased from GE Medical Life Sciences (Harrogate, UK), streptomycin and TritonX-100 from Solarbio Co. Ltd. (Beijing, China), and fetal bovine serum (FBS) from Zhejiang Tianhang Biotechnology Co. Ltd. (Hangzhou, China). Dimethyl sulfoxide (DMSO), murine TNF-α, IFN-γ and IL-2 detection kits, concanavalin-A (ConA), and lipopolysaccharides (LPS) were purchased from Beijing Boao Tuoda Technology Co, Ltd. (Beijing, China). Switzerland-Giemsa compound dyeing liquid was obtained from Nanchang Yulu Experimental Equipment Co. Ltd. (Nanchang, China) Red blood cell lysis buffer was purchased from Tiangen Biochemical Technology (Beijing, China) Co. Ltd. (Beijing, China). All other reagents were of analytical grade.

### 3.2. Extraction and Characterization of Polysaccharides

#### 3.2.1. Extraction and Purification

The clean and dry RHWs were pulverized and passed through a 60-mesh sieve, then refluxed twice with 75% ethanol (1:15) in a water bath at 60 °C for 4 h. The final extract was centrifugated, and the precipitate was dried in an oven at 60 °C and then passed through a 60-mesh sieve. Five grams of the RHW powder was soaked in 30 volumes of distilled water for 90 min and extracted twice at 60 °C for 60 min (Shanghai Billing Instrument Co. Ltd., Shanghai, China). The extracts were pooled and concentrated to about 40 mL in the RE-5203 rotary evaporator (Shanghai Yarong Biochemical Instrument Factory, Shanghai, China). After removing proteins by the Sevag method (chloroform: n-butanol = 5:1) [39], we diluted the extract with 4 volumes of 80% ethanol and left it undisturbed at 4 °C overnight. The mixture was centrifuged, and the precipitate was harvested and lyophilized to obtain the crude RHWPs.

The following experimental conditions were tested: extraction temperature of 20–100 °C, extraction time 20–100 min, liquid/solid ratio 10–50 mL/g, ethanol concentration 60–100%, number of extractions 1–5, and soaking time of 30–150 min.

#### 3.2.2. Quantification of RHWPs

A 200 μg/mL stock solution of glucose was prepared in distilled water and serially diluted to 0, 20, 40, 60, 80, 100, and 120 μg/mL. Two milliliters of each standard were mixed with 1 mL 5% phenol and 5 mL concentrated sulfuric acid [40,41]. The mixture was left undisturbed for 20 min, and the absorbance at 490 nm was measured using a UV-5100 UV visible spectrophotometer (Shanghai Yuanxie Instrument Co., Ltd., Shanghai, China). The absorbance (Y) was plotted on the ordinate axis, and the corresponding concentration (X) on the abscissa axis. The regression equation obtained was Y = 0.0148X + 0.0004, and the correlation coefficient was *R^2^* = 0.9997. The dried RHWP was dissolved in 50 mL distilled water, and then diluted 500 times. The absorbance of 1 mL sample solution was measured as described above. The concentration of RHWP was determined using the regression equation, and the extraction rate was calculated by the following equation:

RHWPs rate (%) = (C × V × D)/(W × 10^6^) × 100% (1)

where C is the concentration of RHWPs (µg/mL), V is the total volume of the extraction solution (mL), D is the dilution factor, and W is the weight of RHW.

#### 3.2.3. Uniform Design Experimentation

The uniform design (U_6_(6^4^)) was used on a single-factor basis to optimize the extraction process. Extraction temperature (A), extraction time (B), liquid/solid ratio (C), and soaking time (D) were the 4 factors that were tested. The factors and levels of the independent variables are shown in Table 8.

#### 3.2.4. Purification of RHWPs

The crude RHWPs were dissolved in deionized water and purified by D101 macroporous adsorption resin to eliminate the pigment, and DEAE Sephadex™ A-25 to eliminate other carbohydrates. Ten crude RHWPs aqueous solutions (30 mg/mL) were loaded onto a DEAE-52-cellulose chromatography column (Ø 2.5 cm × 40 cm) and eluted with deionized water to obtain purified RHWPs.

#### 3.2.5. FTIR Spectrometry

The RHWP powder was mixed with KBr (1:50) and homogenized with an agate mortar into a uniform paste [42,43]. After compression, infrared spectrum scanning was performed at 4000~400 cm^−1^ using an FTIR spectrometer (Thermo Fisher Scientific Co., Ltd., Shanghai, China).

#### 3.2.6. GPC Analysis

A certain amount of sample was dissolved in ultrapure water and then mechanically stirred overnight. After that, the sample solution was filtered through 0.22 μm microporous membrane filters, and the filtrate was stored until the subsequent use. Molecular weights were determined using GPC (gel permeation chromatography; Wyatt Technologies, Santa Barbara, CA, USA). The operating conditions are as follows: mobile phase, water + 0.02% NaNO3; column, OHpak series SB-806 and 804; flow rate, 1 mL/min; column temperature, 40 °C; and injection volume, 500 μL.

#### 3.2.7. Analysis of Monosaccharide Compositions

An appropriate amount of the sample was weighed in a 100-mL Erlenmeyer flask and mixed with water. Then, 5 mL each of the zinc acetate and potassium ferrocyanide solutions were slowly added. The solution was shaken at room temperature for 1 h, centrifuged, and filtered with dry filter paper. The pellet was dissolved in the required volume of an appropriate solvent. One milliliter of the sample was mixed with 1.0 mL of 4 mol/L trifluoroacetic acid solution and then hydrolyzed at 120 °C for 120 min. The sample was dried on water bath at 70 °C, and then under nitrogen gas stream. The monosaccharide sample (obtained after hydrolysis and drying) was mixed with anhydrous methanolic solution of 1-phenyl-3-methyl-pyrazolone reagent (0.5 mol/L), and 0.3 mL of 0.3 mol/L NaOH solution was mixed into it. After mixing, the reaction mixture was heated on a water bath at 70 °C for 30 min. After the sample was cooled down to room temperature, 0.5 mL of 0.3 mol/L HCl and 1 mL of chloroform were added. The mixture was shaken, and the chloroform layer was removed; this extraction process was performed 3 times. The water layer was filtered through a 0.22 μm filter and then placed on the machine. Other conditions are as follows: column, Thermo C18 column (4.6 mm × 250 mm, 5 μm); mobile phase, 0.1 mol/L phosphate buffer (pH 6.7): acetonitrile (ratio = 83:17 (*v/v*)); flow rate, 1.0 mL/min; column temperature, 25 °C; injection volume, 10 μL; and wavelength, 250 nm.

### 3.3. In Vitro Antitumor Activity

#### 3.3.1. CCK-8 Assay

S180 cells were seeded in 96-well plates at the density of 1 × 10^4^ cells/100 μL/well, and after a 24 h incubation were cultured with 10 µL RHWPs (0–8 mg/mL) for 24 h at 37 °C under 5% CO_2_, 4 well replicates for each concentration of RHWPs. Ten microliters of CCK-8 reagent was added and the cells were incubated for 4 h. After dissolving the formazan crystals with DMSO, the optical density of the wells was measured at 450 nm using a microplate reader (Bio-Rad Laboratories) [44]. The cell growth inhibition rate was calculated as follows:

Inhibition rate (%) = (1 − OD/OD_0_) × 100 (2)

where OD and OD_0_ indicate the absorbance of treated and untreated cells, respectively.

#### 3.3.2. Florescence Microscopy

S180 cells were seeded in 6-well plates at the density of 1 × 10^6^ cells/mL/well, and treated with the suitable concentration of RHWPs for 72 h. The cells were harvested by centrifuging at 1000 rpm for 10 min, washed once with PBS, and resuspended in PBS at 10^6^ cells/mL. The cell suspension (100 µL) was stained with 10 μL acridine orange (AO; 100 μg/mL) and 10 μL propidium iodide (PI; 100 μg/mL). The stained cells were mounted on a clean glass slide with a coverslip and immediately observed under a fluorescence microscope (Life Technologies, Carlsbad, CA, USA) to detect live (green), apoptotic (orange), and necrotic (red) cells [11].

### 3.4. In Vivo Anti-Tumor Activity

#### 3.4.1. Establishment of Tumor Model and Treatment Regimen

Fifty Kunming mice (25 males and females each, 3–5 weeks old, weighing 20 ± 2 g) were obtained from Southwestern University Rongchang Campus Experimental Animal Center (Chongqing, China). All animal procedures were performed as per internationally accepted principles mentioned in the Guidelines for Keeping Experimental Animals issued by the government of China and approved by the IACUC, Southwest University (no. 2019122418). All mice were acclimatized for 2 days before the experiment. As previously described by Haiquan et al. [45], 2 × 10^5^ S180 cells (0.2 mL of the l × 10^6^ cells/mL suspension) were injected subcutaneously into the right forelimb axilla of each mouse. After 24 h, 50 mice were randomly divided into the control (saline, 0.2 mL/10 g; ig); cyclophosphamide (50 mg/kg, 2 days, ii; saline, 0.2 mL/10 g; 8 d, ig); and the low-, medium-, and high-dose RHWP (50, 150, 450 mg/kg, ig) groups (10 per group). The drugs were administrated once a day starting 24 h after tumor cell inoculation for 10 days.

#### 3.4.2. Gross and Histological Examination of Tumors

The mice were sacrificed 24 h after the last drug administration by cervical dislocation, and the tumors were dissected and weighed. The tumor inhibition rate (%) was calculated as follows:

Tumor inhibition rate (%) = (W_0_ − W_1_)/W_0_ × 100% (3)

where W_0_ and W_1_ are the average tumor weights of control and test groups, respectively.

The tumors were fixed in formalin for 72 h, and paraffin sections were prepared at the Rongchang People’s Hospital (Chongqing, China). The tissue sections were stained with hematoxylin and eosin and observed under a light microscope (UOP, USA) at 100× and 400× magnification to record the pathological changes.

#### 3.4.3. ELISA

The mice were bled retro-orbitally 24 h after the last drug administration, and the blood samples were left undisturbed at 37 °C for 30 min and then at 4 °C for 1 h. The coagulated blood was centrifuged at 3000 rpm for 15 min, and serum was collected and assayed according to the ELISA test kit instructions.

#### 3.4.4. Lymphocyte Proliferation Assay

The spleen was aseptically removed into cold RPMI 1640 in a sterile glass plate, and gently homogenized using the ribbed surface of the syringe handle [24]. The homogenate was filtered through a three-layer nylon mesh into a centrifuge tube, and 2 mL RBC lysis buffer was added, followed by 3 mL RPMI 1640. The cells were washed twice at 1000 rpm for 5 min and resuspended in complete medium (with 20% FBS) to 2 × 10^6^/mL. The spleen cells were seeded in a 96-well plate at a density of 2 × 10^5^ cells/100 μL/well, and 100 µL serum-free media with ConA (final concentration 5 μg/mL) or LPS (final concentration 10 μg/mL) was added per well (triplicate wells for each). The cells were cultured at 37 °C under 5% CO_2_ for 48 h, and additionally for 4 h with 20 μL CCK-8. The absorbance at 450 nm was measured using a microplate reader, and the lymphocyte proliferation capacity was calculated as follows:

Lymphocyte proliferation capacity = OD_1_/OD_0_ (4)

where OD_0_ and OD_1_ are the OD values of control and experimental wells, respectively.

#### 3.4.5. NK Cell Killing Assay

The target YAC-1 cells were passaged 24 h before the test, washed thrice with serum-free RPMI 1640, and resuspended to 1 × 10^5^ cells/mL in complete medium (with 10% FBS). The spleen cell suspension was prepared as above at the density of 5 × 10^6^ cells/mL. The target and effector cells were mixed in the ratio of 50:1 (100 µL of each) and seeded into 96-well culture plates (natural release wells) [24]. In addition, 100 μL target cells were seeded with 2% Triton X-100 as positive control (maximum release wells). The cells were cultured at 37 °C under 5% CO_2_ for 4 h, and the plates were centrifuged at 1500 rpm for 5 min. The supernatants were aspirated, and 100 μL from each well was transferred to a 96-well culture plate. After adding 100 μL lactate dehydrogenase (LDH) substrate solution and incubating the mixture for 3 min, we added 30 μL of 1 M citrate solution to each well to terminate the reaction. The OD value was measured at 570 nm using a microplate reader, and the killing activity of NK cells was calculated as

NK cell killing activity (%) = (OD_1_ − OD_0_)/(OD_2_ − OD_0_) × 100% (5)

where OD_0_, OD_1_, and OD_2_ are the OD values of natural release well, reaction well, and maximum release well, respectively. Triplicate wells were set up for each reaction.

#### 3.4.6. Phagocytosis Assay

Twenty-four hours before the last drug administration, 1 mL 5% chicken RBCs were injected into the peritoneal cavity of each mouse, followed by 2 mL physiological saline 24 later. The euthanized mice were immersed in 75% ethanol for 2 min, and their abdominal cavities were opened. The abdominal fluid was collected and dropped on glass slides, then incubated for 30 min at 37 °C. The cells were fixed with 1:1 acetone/methanol (*v/v*) for 5 min, stained with 4% Giemsa for 3 min, rinsed with running water, and then air-dried. The stained PMs were counted under a microscope [26]. The phagocytosis rate and phagocytic index of the PMs were calculated as follows:

Phagocytosis rate (PP) = (number of cells phagocytizing chicken RBCs per 100 phagocytic cells/100) × 100% Phagocytic index (PI) = total number of chicken RBCs engulfed by 100 phagocytic cells/100(6)

#### 3.4.7. NO production Assay

The standard curve of NO was first established using the Griess method [46]. Briefly, 0.69 mg NaNO_2_ was dissolved in 10 mL medium to obtain a 1 mM stock solution, which was then serially diluted to the range of 0 to 100 μM. The dilutions were pipetted into a 96-well cell culture plate at 100 μL/well (triplicate wells for each dilution), and equivalent volume of Griess reagent was added. After incubating for 10 min in the dark, the absorbance at 540 nm was measured with a microplate reader. The concentration of NO was plotted as the abscissa and the absorption as the ordinate. The abdominal cavities of the tumor-bearing mice were aseptically opened as described above, and washed with 10 mL chilled D-Hanks solution. The peritoneal fluid was collected into 50 mL tubes and centrifuged at 4 °C for 10 min (1000 rpm). After washing twice with D-Hanks solution, the PMs were resuspended in RPMI-1640 at 1 × 10^6^/mL, and seeded in 24-well plates at the same density. The cells were cultured at 37 °C under 5% CO_2_ for 48 h, and 100 μL supernatant was aspirated from each well and transferred to a 96-well culture plate. Equal volume of Griess reagent was added and the reaction was performed as described. The NO content of the samples was determined using the standard curve.

### 3.5. Statistical Analysis

All data are presented as mean ± SD. *p <* 0.05 was considered statistically significant. All analysis was performed using Excel (2016), SPSS 22.0 and Prism 5 (Chicago, IL, USA).

## 4. Conclusions

The optimum parameters for hot water RHWP extraction were as follows: 40 min extraction time, liquid/solid ratio 30 mL/g, 100 min soaking time, two extraction cycles, 100% ethanol concentration, and extraction temperature of 80 °C. RHWPs repressed the proliferation of S180 cells and induced apoptosis in vitro, and inhibited the growth of ascites tumors in mice by accelerating tumor cell apoptosis and necrosis. Mechanistically, mid- and high-dose RHWPs were found to collectively promote the anti-tumor immune response by increasing TNF-α, IFN-γ, and IL-2 secretion; T and B cell proliferation; and NK and macrophage effector functions. Thus, these data provide a scientific basis for the potential application of RHWPs as a novel candidate anti-tumor agent.

## Figures and Tables

**Table 1:** Uniform design and test results.

Number	Extraction Temperature (°C)	Extraction Time (min)	Liquid/Solid Ratio (mL/g)	Soaking Time (min)	Extraction Rate (%)
1	1 (50)	2 (50)	3 (40)	6 (150)	33.95
2	2 (60)	4 (70)	6 (55)	5 (140)	32.89
3	3 (70)	6 (90)	2 (35)	4 (130)	35.08
4	4 (80)	1 (40)	5 (50)	3 (120)	37.68
5	5 (90)	3 (60)	1 (30)	2 (110)	38.97
6	6 (100)	5 (80)	4 (45)	1 (100)	37.85

**Table 2:** Analysis of variance.

Project	Sum of Square	Degree of Freedom	Mean Square	F	P
Return Residual Total	117.871 1.150 119.021	3 2 5	39.290 0.575	68.340	0.014

**Table 3:** The molecular weight distribution of RHWPs (Mn: the number average molecular weight; Mw: the average molecular weight; PD: the polydispersity index (PD = Mw/Mn)).

Analysis Item	Index	Peak 1	Peak 2
Molecular weight	PD (Mw/Mn)	3.929	1.083
	Mn (kDa)	58.19	4.619
	Mw (kDa)	2.286 × 10^2^	5.001

**Table 4:** Mass ratio and the molar ratio of each monosaccharide of RHWPs.

Monosaccharide	Mass Ratio	Molar Ratio
Mannose	686.68	3.81
Ribose	57.07	0.38
l-Rhamnose monohydrate	318.68	1.94
Glucuronic acid	242.65	1.25
Galacturonic acid	598.00	3.08
Glucose	27,755.21	154.06
Galactose	6108.74	33.91
Xylose	98.58	0.66
Arabinose	1385.43	9.23
Fucose	161.74	0.99

**Table 5:** Growth inhibition of S180 cells (** indicates that the difference is extremely significant compared with the blank group (*p <* 0.01)).

Concentration (mg/mL)	OD	Inhibition Rate (%)
0	1.109 ± 0.002	-
0.125	0.979 ± 0.026 **	11.74
0.25	0.934 ± 0.011 **	15.78
0.5	0.731 ± 0.009 **	34.12
1	0.699 ± 0.014 **	36.96
2	0.673 ± 0.004 **	39.37
4	0.585 ± 0.058 **	47.31
6	0.440 ± 0.036 **	60.33
8	0.401 ± 0.026 **	63.85

**Table 6:** Inhibition of S180 tumors by RHWPs (significant differences from the control groups are designated as * *p* < 0.05 and ** *p* < 0.01 (*n* = 10)).

Group	Dose (mg/kg)	Tumor Weight (g)	Inhibition Rate (%)
Control	-	0.4227 ± 0.1332	-
CTX	50	0.1150 ± 0.0471 **	72.79
RHWPs-Low	50	0.3053 ± 0.1059 *	27.77
RHWPs-Mid	150	0.1537 ± 0.0573 **	63.63
RHWPs-High	450	0.2241 ± 0.1336 **	46.98

**Table 7:** Effect of RHWPs on tumor cell-killing activity of NK cells. (There is no significant differences from the control groups (n = 10)).

Group	Dose (mg/kg)	Natural Release Well (OD)	Reaction Well (OD)	Maximum Release Well (OD)	Killing Activity (%)
Control	-	0.244 ± 0.010	0.254 ± 0.004	0.518 ± 0.008	3.65
CTX	50	0.162 ± 0.026	0.270 ± 0.002	0.434 ± 0.002	39.71
RHWPs-Low	50	0.090 ± 0.001	0.153 ± 0.027	0.675 ± 0.022	10.77
RHWPs-Mid	150	0.097 ± 0.006	0.170 ± 0.001	0.427 ± 0.085	22.12
RHWPs-High	450	0.024 ± 0.004	0.172 ± 0.001	0.521 ± 0.012	29.78

**Table 8:** The levels of variables employed in the uniform design.

Number	Extraction Temperature (°C)	Extraction Time (min)	Liquid/Solid Ratio (mL/g)	Soaking Time (min)
1	1 (50)	2 (50)	3 (40)	6 (150)
2	2 (60)	4 (70)	6 (55)	5 (140)
3	3 (70)	6 (90)	2 (35)	4 (130)
4	4 (80)	1 (40)	5 (50)	3 (120)
5	5 (90)	3 (60)	1 (30)	2 (110)
6	6 (100)	5 (80)	4 (45)	1 (100)

## Data Availability

Not applicable.

## Sample Availability

Samples of the compounds are available from the authors.

## Abbreviations

RHWPs	polysaccharides from the root of Henry wood betony
FACS	fluorescence activated cell sorting
FITC	fluorescein isothiocyanate
NK	natural killer cells
PP	phagocytosis rate
PI	phagocytic index
IL-2	interleukin-2
TNF-α	tumor necrosis factor alpha
IFN-γ	interferon-γ
LPS	lipopolysaccharide
ConA	concanavalin A
IR	infrared
PM	peritoneal macrophages
